# DNA barcodes identify Central Asian *Colias* butterflies (Lepidoptera, Pieridae)

**DOI:** 10.3897/zookeys.365.5879

**Published:** 2013-12-30

**Authors:** Juha Laiho, Gunilla Ståhls

**Affiliations:** 1 Persövägen 148, FI-10600 Ekenäs, Finland; 2 Finnish Museum of Natural History, Zoological museum, PO Box 17, FI-00014 University of Helsinki, Finland

**Keywords:** Barcoding, COI, *Colias*, Central-Asia, RpS2

## Abstract

A majority of the known *Colias* species (Lepidoptera: Pieridae, Coliadinae) occur in the mountainous regions of Central-Asia, vast areas that are hard to access, rendering the knowledge of many species limited due to the lack of extensive sampling. Two gene regions, the mitochondrial COI ‘barcode’ region and the nuclear ribosomal protein RpS2 gene region were used for exploring the utility of these DNA markers for species identification. A comprehensive sampling of COI barcodes for Central Asian *Colias* butterflies showed that the barcodes facilitated identification of most of the included species. Phylogenetic reconstruction based on parsimony and Neighbour-Joining recovered most species as monophyletic entities. For the RpS2 gene region species-specific sequences were registered for some of the included *Colias* spp. Nevertheless, this gene region was not deemed useful as additional molecular ‘barcode’. A parsimony analysis of the combined COI and RpS2 data did not support the current subgeneric classification based on morphological characteristics.

## Introduction

The use of a standardized gene region, i.e. a 650 bp fragment of the 5’-region of the mitochondrial cytochrome *c* oxidase subunit I (hereafter COI), as a DNA barcode (Hebert et al. 2003), to facilitate identification of biological specimens, as well as for calling attention to possible new species has generated a steadily increasing number of DNA barcoding studies of invertebrates (Taylor and Harris 2012), and particularly of Lepidoptera (see www.lepbarcoding.org). While the utility of DNA barcoding as an investigative tool has gained much support, there still remain a number of problems related to the use of a single DNA sequence as a taxon barcode. Several studies on Lepidoptera have shown that species may be polymorphic and/or share haplotypes (Nice et al. 2002, Wahlberg et al. 2003, Elias et al. 2007, Schmidt and Sperling 2008), so that identifications may become less reliable. Additionally, it has been shown that incomplete lineage sorting or mitochondrial introgression could obscure the delimitation of closely related taxa (Tautz et al. 2003, Zakharov et al. 2009). Using one or a few specimens as representatives of a species indeed provides us with little information about their intraspecific variation, particularly for widely distributed species (e.g. Funk and Omland 2003, Seberg et al. 2003, Sperling 2003).

## The genus *Colias*

The butterfly genus *Colias* Fabricius, 1807 is a genus of the family Pieridae (subfamily Coliadinae), comprising about 85 species. Most of its species have a limited distribution in the Arctic and Alpine regions of the Holarctic realm, but two species occur in the Afrotropical and seven are known from the Neotropical regions (Verhulst 2000). A few species are widely distributed and common, such as the Palaearctic *Colias erate* (Esper, 1805) and *Colias croceus* (Geoffroy, 1785), and the Nearctic *Colias eurytheme* Boisduval, 1852 and *Colias philodice* Godart, 1819. As a consequence, these taxa are frequently used in ethological, ecological and genetic research (e.g. Pollock et al. 1998, Wang and Porter 2004, Porter and Levin 2010). *Colias erate* and *Colias croceus* are a species pair where only typical specimens can be reliably distinguished morphologically, and members of these species are known to frequently hybridize (e.g. Dinca et al. 2011 and references therein). Lukhtanov et al. (2009) indicated that mitochondrial introgression was a likely explanation for the shared barcodes they registered between these sympatric taxa. The Nearctic taxa *Colias eurytheme* and *Colias philodice* are broadly sympatric sister species that hybridize frequently and that likely share a significant portion of their genomes through introgression (e.g. Wang and Porter 2004, Porter and Levin 2010). Verhulst (2000) illustrated hybrid individuals of six species of *Colias* from the Palaearctic region, including *Colias croceus*.

The Central Asian mountainous regions harbour nearly half of all *Colias* species. The distribution, ecology and taxonomy are still incompletely documented for most of these species, mainly due to their remote occurrences (Verhulst 2000). Central Asian *Colias* species occurring in remote mountainous areas that are hard to access have been far less studied than their North American or European congeners. An important part of the older material that exists in museum collections worldwide (e.g. from Tibet) originates from early collecting expeditions in the late 19^th^ and early 20^th^ centuries. Important material was, however, also collected within the former Soviet Union during 20^th^ century. Fieldwork in Central-Asia has subsequently become less complicated, and thus new material is again available for research. As a result of this, new species such as *Colias aegidii* Verhulst, 1990 and *Colias adelaidae* Verhulst, 1991, have been described, as well as a number of new subspecies. Despite an increasing research effort on Central Asian *Colias* species there are as yet no published studies on their phylogenetic relationships.

The first contribution to the species classification of *Colias* was given by Berger (1986), who used a few morphological characters to establish a comprehensive subgeneric classification, comprising the subgenera *Colias* Fabricius, 1807, *Neocolias* Berger, 1986, *Eucolias* Berger, 1986, *Eriocolias* Watson, 1895, *Palaeocolias* Berger, 1986, *Similicolias* Berger, 1986, *Scalidoneura* Butler, 1869 and *Paracolias* Berger, 1986. Later, Ferris (1993) used 84, mainly morphological, characters to reconstruct a phylogeny of all North American *Colias* species known at that time, which was the first species phylogeny within the genus *Colias*. The first contribution to the knowledge of the molecular phylogenetic relationships of the North American *Colias* species was made by Pollock et al. (1998), who studied a number of *Colias* species using a 333 bp sequence fragment of the mtDNA COI gene. They found some small differences between species classified in the subgenera *Neocolias* and *Eriocolias*, thus supporting Berger’s (1986) separation of *Neocolias* from *Eriocolias*. Pollock et al. (1998) also noted that even though *Colias* is a speciose genus, this was not mirrored in the COI sequence diversity. Wheat and Watt (2008) studied the molecular phylogenetic relationships of North American *Colias* taxa using mitochondrial gene sequences (ribosomal 12S and 16S rRNA, Leu2 and Val tRNA and COI + II). Their results showed that the COI sequences only allowed identification of some of the taxa supported by the full data set used in their study. The results of their study further suggested that species radiations within *Colias* are comparatively young as compared with those of related pierid butterflies, since molecular divergences among species were small. Based on molecular data Brunton (1998) studied the phylogenetic relationships of the 12 *Colias* species occurring in Europe. He recovered three monophyletic groups largely corresponding to geographical distributions. He concluded that the Scandinavian species appeared to be the oldest in Europe, sharing a common ancestor with *Colias* species from the USA. According to Brunton (1998) the European *Colias* species radiated from Scandinavia to the rest of Europe forming an eastern clade and a western clade. As with Pollock et al. (1998), the results did not agree with Berger’s (1986) subgeneric classification.

The aim of the present study was to test the usefulness of COI barcodes for species identification of a broad representation of Central Asian *Colias* species, including nine *Colias* species overlapping with Lukhtanov et al.’s (2009) study, and 19 species not previously barcoded. In addition, we wanted to elucidate the informativeness of the RpS2 gene region that Wahlberg and Wheat (2008) found informative for lepidopteran phylogenetic relationships. We tested the nuclear ribosomal protein gene RpS2 as a potential complementary barcode region for *Colias* and for use in a combined analysis with COI for testing the current subgeneric classification of the species in the present study. We also contrasted our COI barcodes against a larger set of COI barcodes of *Colias* taxa available from GenBank (GB).

## Materials and methods

### Study area and taxon sampling

This study includes material from the mountain regions of Kirgizistan, Tadzhikistan, northern Afghanistan, northern Pakistan and India (e.g. mountain ranges Tian Shan, Hindu Kush, Karakorum, Himalaya) and the mountain regions in the Chinese provinces Qinghai, Gansu, Sichuan, Yunnan and the autonomous regions Tibet and Xinjiang Uygur. The *Colias* fauna of these Central Asian regions comprises about 34 species (Verhulst 2000) while the species number for Central Asia in broad sense is over 40 species.

The taxon sampling aimed to cover as many of the *Colias* species from this area as possible. Additionally, a few *Colias* species occurring in adjacent territories (e.g. Buryatia) were also available for molecular study. Whenever possible, several individuals of each species were analysed to assess intraspecific variation. The available specimens used for molecular study consisted of a total of 56 adult specimens covering 27 species of Central Asian *Colias* and two *Colias* species from adjacent territories (Table 1). The specimens are preserved as DNA voucher specimens and labelled accordingly, to be deposited in the collections of the Zoological Museum of Finnish Museum of Natural History, Helsinki, Finland (MZH) (DNA voucher specimens MZH_JL1-JL71). Species identifications were verified by JL based on easily recognizable diagnostic characters using the monograph by Verhulst (2000), while the taxonomy is according to Grieshuber and Lamas (2007). Additionally, we used 35 COI barcode sequences (17 species) of Palaearctic *Colias* species obtained from GB, as listed in Table 2.

**Table 1.** List of specimens used for molecular analyses including GenBank accession numbers.

Species	Sex	Locality and date	Lab code	COI accession number	RpS2 accession number
**subgenus *Colias* Fabricius, 1807**					
*Colias hyale* (Linnaeus, 1758) *irkutskana* Stauder, 1923	male	Russia, SW Transbaikalia, Buryatia, Selenga river district, Gusinoye Ozero village env., steppe rivulet valley, 7.6.2003	MZH_JL35	HE775142	HE775198
*Colias hyale* (Linnaeus, 1758) *irkutskana* Stauder, 1923	male	Russia, SW Transbaikalia, Buryatia, Selenga river district, Gusinoye Ozero village env., steppe rivulet valley, 7.6.2003	MZH_JL44	HE775143	HE775199
**subgenus *Eriocolias* Berger, 1986**					
*Colias adelaidae adelaidae* Verhulst, 1991	male	China, Gansu, Xia-He, 3400 m, 35°11'N, 102°31'E, 25.6.2004	MZH_JL61	HE775187	HE775243
*Colias alpherakii alpherakii* Staudinger, 1882	female	Kyrgyzstan, Alai mts., 4 km SE Tengizbai pass, 3400 m, 3.7.2001	MZH_JL37	HE775169	HE775225
*Colias alpherakii alpherakii* Staudinger, 1882	female	Kyrgyzstan, Alai mts., 4 km SE Tengizbai pass, 3400 m, 3.7.2001	MZH_JL51	HE775180	HE775236
*Colias berylla berylla* Fawcett, 1904	male	China, S Tibet, Himalaya Mts., Lablungla pass, 4800 m, 18–22.7.2001	MZH_JL48	HE775178	HE775234
*Colias berylla berylla* Fawcett, 1904	male	China, Tibet, Lhodak, 4600 m, 15.7.2002	MZH_JL55	HE775182	HE775238
*Colias christophi christophi* Grum Grshimailo, 1885	female	Tadjikistan, Turkestanskyi Mts., Kumbel pass, 3000 m, July 2002	MZH_JL45	HE775175	HE775231
*Colias christophi helialaica* Schulte, 1988	male	Kyrgyzstan, Alai Mts., W end of Tengizbai pass, 3700 m, 5–6.7.2001	MZH_JL67	HE775192	HE775246
*Colias cocandica cocandica* Erschoff, 1874	male	Kyrgyzstan, Suusamyr Mt. r., Alabel pass, 3200 m, 10.7.2002	MZH_JL43	HE775174	HE775230
*Colias cocandica hinducucica* Verity, 1911	male	Tajikistan, E Pamir, Ak-Buura Mts., 4250 m, 14–15.7.2003	MZH_JL34	HE775168	HE775224
*Colias cocandica pljushtchi* Verhulst, 2000	male	Kyrgyzstan, Sary Dzhaz riv. bas., Kaindy-Ketta mts., Tashkoro village, 3000 m 10.7.2003	MZH_JL19	HE775160	HE775216
*Colias eogene* C. et R. Felder, [1865] *elissa* Grum Grshimailo, 1890	male	Kyrgyzstan, W end of Tengizbai pass, 3700 m, 5–6.7.2001	MZH_JL1	HE775144	HE775200
*Colias eogene* C. et R. Felder, [1865] *elissa* Grum Grshimailo, 1890	male	Kyrgyzstan, W end of Tengizbai pass, 3700 m, 5–6.7.2001	MZH_JL40	HE775171	HE775227
*Colias fieldii* Ménétriés, 1855 *chinensis* Verity, 1909	male	China, Sichuan, Zhangia, 3000 m, 32°47'N, 103°36'E, 6.6.2002	MZH_JL50	HE775179	HE775235
*Colias fieldii* Ménétriés, 1855 *chinensis* Verity, 1909	female	China, Gansu, Shin-Long-Shan, 2800 m, 35°48'N, 103°59'E, 29.6.2004	MZH_JL60	HE775186	HE775242
*Colias grumi grumi* Alphéraky, 1897	female	China, Gansu, Altun Shan, road from Aksay to Danjing pass, 2500–2800 m, 22–23.7.2002	MZH_JL54	HE775197	-
*Colias heos heos* (Herbst, 1792)	male	Russia, SW Transbaikalia, Buryatia, Selenga river district, Gusinoye Ozero village env., steppe rivulet valley, 1.7.2003	MZH_JL39	HE775170	HE775226
*Colias heos heos* (Herbst, 1792)	male	Russia, SW Transbaikalia, Buryatia, Selenga river district, Gusinoye Ozero village env., steppe rivulet valley, 1.7.2003	MZH_JL46	HE775176	HE775232
*Colias lada lada* Grum Grshimailo, 1891	male	China, Sichuan, Maningano surr., 31°56'N, 99°12'E, 4500 m, 15.6.2002	MZH_JL7	HE775150	HE775206
*Colias lada lada* Grum Grshimailo, 1891	male	China, Sichuan, Maningano surr., 31°56'N, 99°12'E, 4500 m, 15.6.2002	MZH_JL27	HE775165	HE775221
*Colias ladakensis* Felder, 1865 *seitzi* Bollow, 1939	male	China, SW Tibet, Himalaya Mts., 100km W Paryang, 4650–5000 m, 13.6.2004	MZH_JL4	HE775147	HE775203
*Colias ladakensis* Felder, 1865 *seitzi* Bollow, 1939	male	China, SW Tibet, Himalaya Mts., 100km W Paryang, 4650–5000 m, 13.6.2004	MZH_JL57	HE775183	HE775239
*Colias marcopolo marcopolo* Grum Grshimailo, 1888	male	Tadjikistan, E Pamir, Dunkeldyk Lake, 4400 m, 25.7.2003	MZH_JL30	HE775166	HE775222
*Colias marcopolo marcopolo* Grum Grshimailo, 1888	male	Tadjikistan, E Pamir, Dunkeldyk Lake, 4400 m, 25.7.2003	MZH_JL33	HE775167	HE775223
*Colias marcopolo marcopolo* Grum Grshimailo, 1888	male	Tadjikistan, E Pamir, Dunkeldyk Lake, 4400 m, 25.7.2003	MZH_JL41	HE775172	HE775228
*Colias montium montium* Oberthür, 1886	male	China, Sichuan, Maningano surr., 31°55'N, 99°12'E, 4000 m, 9–18.6.2004	MZH_JL59	HE775185	HE775241
*Colias nebulosa* Oberthür, 1894 *sungpani* Bang-Haas, 1927	male	China, Sichuan, Maningano surr., 31°56'N, 99°12'E, 4500 m, 15.6.2002	MZH_JL9	HE775152	HE775208
*Colias nebulosa* Oberthür, 1894 *sungpani* Bang-Haas, 1927	male	China, Sichuan, Maningano surr., 31°56'N, 99°12'E, 4500 m, 15.6.2002	MZH_JL24	HE775162	HE775218
*Colias nebulosa* Oberthür, 1894 *sungpani* Bang-Haas, 1927	male	China, Sichuan, Maningano surr., 31°56'N, 99°12'E, 4500 m, 15.6.2002	MZH_JL26	HE775164	HE775220
*Colias nina* Fawcett, 1904 *hingstoni* Riley, 1923	male	China, SW Tibet, Himalaya Mts., 60 km S Saga, 4600–5000 m, 7–8.6.2004	MZH_JL53	HE775181	HE775237
*Colias nina* Fawcett, 1904 *hingstoni* Riley, 1923	male	China, SW Tibet, Himalaya Mts., Lablongla pass, 4800 m, 5.6.2004	MZH_JL58	HE775184	HE775240
*Colias regia regia* Grum Grshimailo, 1887	male	Kyrgyzstan, Kaindy-Ketta Mt. r., Kumar pass, 3200 m, 12.7.2003	MZH_JL8	HE775151	HE775207
*Colias regia regia* Grum Grshimailo, 1887	male	Kyrgyzstan, Kaindy-Ketta Mt. r., Kumar pass, 3200 m, 12.7.2003	MZH_JL42	HE775173	HE775229
*Colias romanovi romanovi* Grum Grshimailo, 1885	male	Kyrgyzstan, Alai mts., 4 km SE Tengizbai pass, 3400 m, 7–8.7.2001	MZH_JL3	HE775146	HE775202
*Colias romanovi romanovi* Grum Grshimailo, 1885	male	Kyrgyzstan, Alai mts., 4 km SE Tengizbai pass, 3400 m, 7–8.7.2001	MZH_JL47	HE775177	HE775233
*Colias sieversi sieversi* Grum Grshimailo, 1887	male	Tadjikistan, Peter I Mts., Ganishob, 2400 m, 17.6.2004	MZH_JL70	HE775195	-
*Colias sifanica sifanica* Grum Grshimailo, 1891	male	China, Gansu, Xia-He, 3400 m, 35°11'N, 102°31'E, 25.6.2004	MZH_JL11	HE775154	HE775210
*Colias sifanica sifanica* Grum Grshimailo, 1891	male	China, Gansu, Xia-He, 3400 m, 35°11'N, 102°31'E, 25.6.2004	MZH_JL64	HE775189	HE775245
*Colias staudingeri* Alphéraky, 1881 *pamira* Grum Grshimailo, 1890	male	Kyrgyzstan, Zaalaisky (Transalai) Mts., Altyn Dara river, 3000 m, 25.7.2000	MZH_JL2	HE775145	HE775201
*Colias staudingeri* Alphéraky, 1881 *pamira* Grum Grshimailo, 1890	male	Kyrgyzstan, Zaalaisky (Transalai) Mts., Altyn Dara river, 3000 m, 25.7.2000	MZH_JL13	HE775156	HE775212
*Colias staudingeri* Alphéraky, 1881 *pamira* Grum Grshimailo, 1890	male	Kyrgyzstan, Zaalaisky (Transalai) Mts., Altyn Dara river, 3000 m, 25.7.2000	MZH_JL23	HE775161	HE775217
*Colias stoliczkana stoliczkana* Moore, 1882	male	India, Jammu Kashmir, Ladakh Range, Markha Valley, Ganda Pass, 4600 m, 12.7.2001	MZH_JL15	HE775158	HE775214
*Colias thisoa* Ménétriés, 1832 *aeolides* Grum Grshimailo, 1890	male	Kyrgyzstan, Sary Dzhaz riv. bas., Kaindy-Ketta mts., Tashkoro village, 3000 m, 10.7.2003	MZH_JL10	HE775153	HE775209
*Colias thisoa* Ménétriés, 1832 *aeolides* Grum Grshimailo, 1890	female	Kyrgyzstan, Sary Dzhaz riv. bas., Kaindy-Ketta mts., Tashkoro village, 3000 m, 10.7.2003	MZH_JL17	HE775159	HE775215
*Colias thisoa* Ménétriés, 1832 *aeolides* Grum Grshimailo, 1890	female	Kyrgyzstan, Sary Dzhaz riv. bas., Kaindy-Ketta mts., Tashkoro village, 3000 m, 10.7.2003	MZH_JL25	HE775163	HE775219
*Colias thrasibulus thrasibulus* Fruhstorfer, 1910	male	China, W Tibet, Mandhata Mt., 4900 m, 15–16.7.2003	MZH_JL14	HE775157	HE775213
*Colias tibetana tibetana* Riley, 1922	male	China, Tibet, Himalaya Mts., Nyalam, 4200 m, 8.7.2003	MZH_JL6	HE775149	HE775205
*Colias tibetana tibetana* Riley, 1922	male	China, SW Tibet, Himalaya Mts., Nyalam, 3700–4200 m, 28–30.6.2004	MZH_JL63	HE775188	HE775244
*Colias wanda wanda* Grum Grshimailo, 1907	male	China, Qinghai, 20km NW of Zhidoi City, 4700–5000 m, 16.7.2000	MZH_JL66	HE775191	-
*Colias wanda wanda* Grum Grshimailo, 1907	male	China, S. Tibet, Cona, 4500–4700 m, 24–25.6.2004	MZH_JL69	HE775194	-
*Colias wiskotti* Staudinger, 1882 *draconis* Grum Grshimailo, 1891	male	Uzbekistan, Chandalas Mts., Chakmksh village, 2600 m, 27.6.2004	MZH_JL71	HE775196	-
*Colias wiskotti* Staudinger, 1882 *hofmannorum* Eckweiler, 2000	male	Iran, Khorasan, 75km SE of Birjand, 2200 m, 18–20.5.2002	MZH_JL68	HE775193	-
*Colias wiskotti* Staudinger, 1882 *separata* Grum Grshimailo, 1888	male	Kyrgyzstan, Alai mts., 4km SE Tengizbai pass, 3400 m, 3.7.2001	MZH_JL65	HE775190	-
**subgenus *Eucolias* Berger, 1986**					
*Colias tyche tyche* (de Boeber, 1812)	male	Russia, East Siberia, Lake Baikal, Khamar-Daban Mts., Slyudyanka river, taiga, 800 m, 14.6.2003	MZH_JL5	HE775148	HE775204
*Colias tyche tyche* (de Boeber, 1812)	male	Russia, East Sayan, Buryatia, Mondy env., Huruma river, 1500 m, 6.6.2002	MZH_JL12	HE775155	HE775211

**Table 2.** List of *Colias* GenBank samples of the COI barcode used in this study.

Species	GenBank accession number
*Colias alpherakii*	FJ663407
*Colias christophi*	FJ663409
*Colias chrysotheme elena*	FJ663410
*Colias chrysotheme elena*	FJ663411
*Colias croceus*	EF457737
*Colias croceus*	FJ663412
*Colias croceus*	GU688507
*Colias croceus*	HQ004279
*Colias croceus*	HQ004282
*Colias eogene*	FJ663415
*Colias eogene*	FJ663416
*Colias erate amdensis*	EF457736
*Colias erate poliographus*	EF457735
*Colias erate poliographus*	EU583852
*Colias erate poliographus*	GU372561
*Colias fieldii*	EF584859
*Colias hyale*	FJ663418
*Colias hyale*	FJ663421
*Colias hyale*	HQ004297
*Colias hyperborea*	EF457739
*Colias marcopolo*	FJ663422
*Colias marcopolo*	FJ663423
*Colias myrmidone*	HQ004303
*Colias phicomone*	HM393178
*Colias regia*	FJ663427
*Colias tamerlana mongola*	FJ663424
*Colias tamerlana mongola*	FJ663425
*Colias tamerlana mongola*	FJ663426
*Colias thisoa thisoa*	FJ663429
*Colias tyche*	FJ663430
*Colias wiskotti chrysoptera*	FJ663431
*Colias wiskotti chrysoptera*	FJ663432
*Colias wiskotti chrysoptera*	FJ663433
*Colias wiskotti wiskotti*	FJ663435
*Colias wiskotti wiskotti*	FJ663436

### Laboratory methods

Total genomic DNA was extracted form 2-5 legs of dried, pinned butterfly specimens using NucleoSpin® Tissue Kit (Machery-Nagel), according to manufacturer’s protocols, and resuspended in 50 µl ultrapure water.

The primer pair LCO-1490 (5’-GGTCAACAAATCATAAAGATATTGG-3’) and HCO-2198 (5’-TAAACTTCAGGGTGACCAAAAAATCA-3’) (Folmer et al. 1994) was used to amplify a ca. 650 bp fragment of the mitochondrial COI gene. The polymerase chain reactions (PCR) were done under the following parameters: initial heating 95 °C for 2 min, following 30 cycles of 94 °C for 30 s, 49 °C for 30 s and 72 °C for 2 min, followed by a final extension of 72 °C for 7 min. The primer pair RpS2 nF (5’-ATCWCGYGGTGGYGATAGAG-3’) and RpS2 nR (5’-ATGRGGCTTKCCRATCTTGT-3’) (Wahlberg and Wheat 2008) was used to amplify a ca. 400 bp fragment of the nuclear RpS2 gene. The PCR were carried out following the PCR cycling profile described in Wahlberg and Wheat (2008): initial heating 95 °C for 7 min, 40 cycles of 95 °C for 30 s, 50 °C for 30 s, 72 °C for 2 min, and a final extension period of 72 °C for 10 min. Sequencing of the double-stranded PCR product was carried out on an ABI PRISM® 377 Automated Sequencer (Applied Biosystems) following manufacturer’s recommendations. All PCR primers were used for sequencing. Sequences were inspected and edited using Sequence Navigator® (Applied Biosystems).

### Sequence analysis

We analysed and clustered our sequence data using parsimony and Neighbour-Joining (NJ) of K2P-distances. We used parsimony and NJ for our newly generated COI sequence dataset, NJ for RpS2 sequences, parsimony for the concatenated COI and RpS2 sequences, and, finally, NJ for the combined COI sequences generated in this study and those in GB. All trees were rooted using *Papilio glaucus* (family Papilionidae) and *Aporia crategi* (Pieridae, subfamily Pierinae) as outgroup taxa.

Parsimony analysis was performed using NONA (Goloboff 1999) and spawn with the aid of Winclada (Nixon 2002), using a heuristic search algorithm with 1000 random addition replicates (mult*1000), holding 10 trees per round (hold/10), max trees set to 10 000 and applying TBR branch swapping. All base positions were treated as equally weighted characters. Nodal support was assessed with bootstrap resampling (1000 replicates) using Winclada (Nixon 2002). MEGA5 (Tamura et al. 2011) was used for NJ clustering using 1000 bootstrap replicates. The Kimura 2-parameter model was used for NJ clustering of the COI sequences, while the Tamura-Nei model with gamma distributed rates was chosen for the RpS2 sequences.

## Results

### Sequences

We obtained a 643 bp COI barcode for 56 *Colias* specimens, and a 409 bp fragment of RpS2 was obtained for 49 specimens (Table 1). A+T content of the COI sequences was 69.22%, and of the RpS2 45.0%. There were 115 parsimony informative sites for COI and 39 for RpS2.

Uncorrected pairwise divergences between ingroup taxa ranged between 1.09 and 4.09% (mean 2.77%) for COI and 0.0–1.7% (mean 1.0%) for RpS2. GenBank accession numbers are given in Table 1. Intraspecific uncorrected distances were up to 1.09% (in *Colias thisoa*) for COI, with specimens of most species differing by less than 4 nucleotide changes.

### Identification: COI vs. RpS2

**Figure 1.**
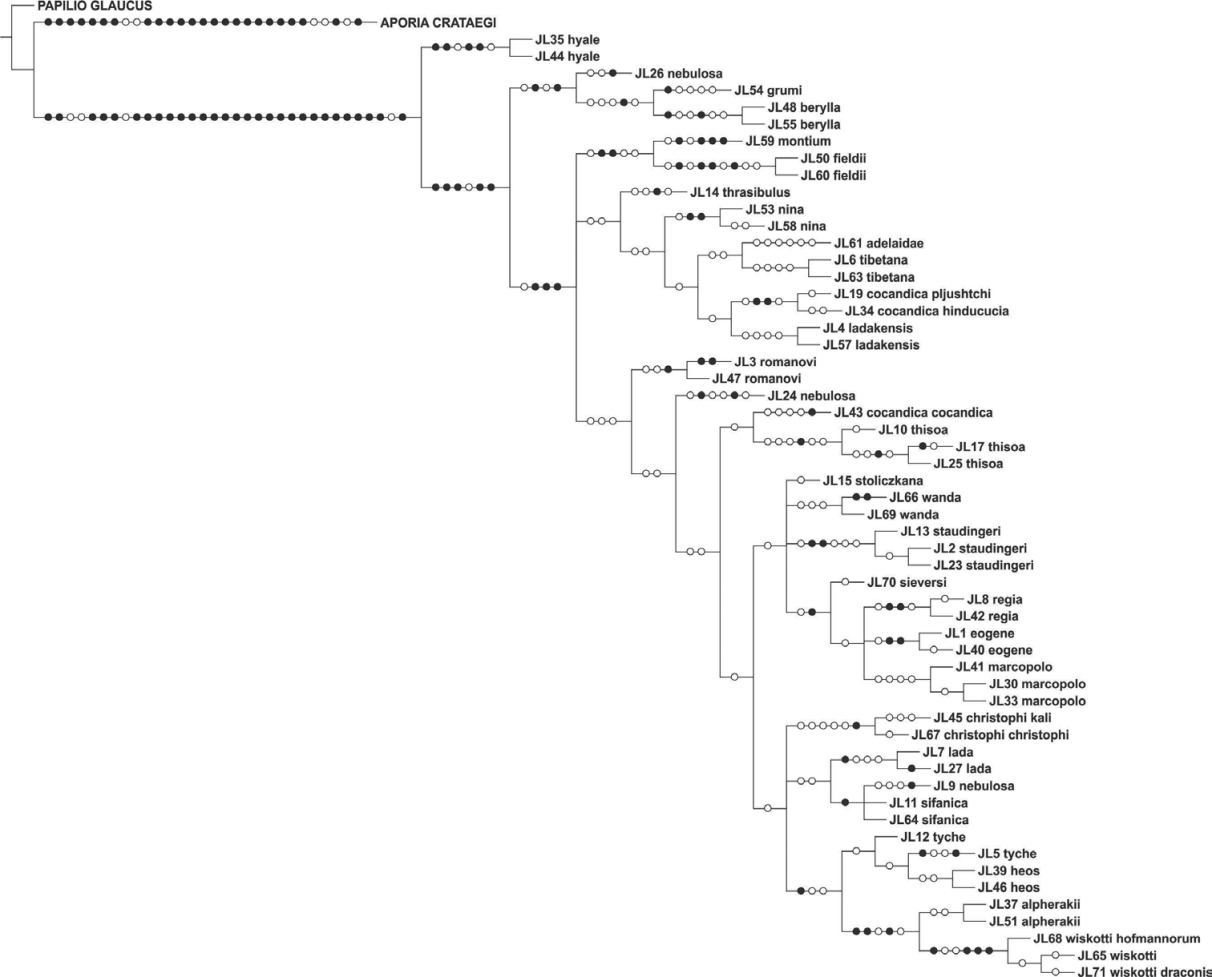
Strict consensus cladogram of *Colias* COI sequences obtained in this study.

**Figure 2.**
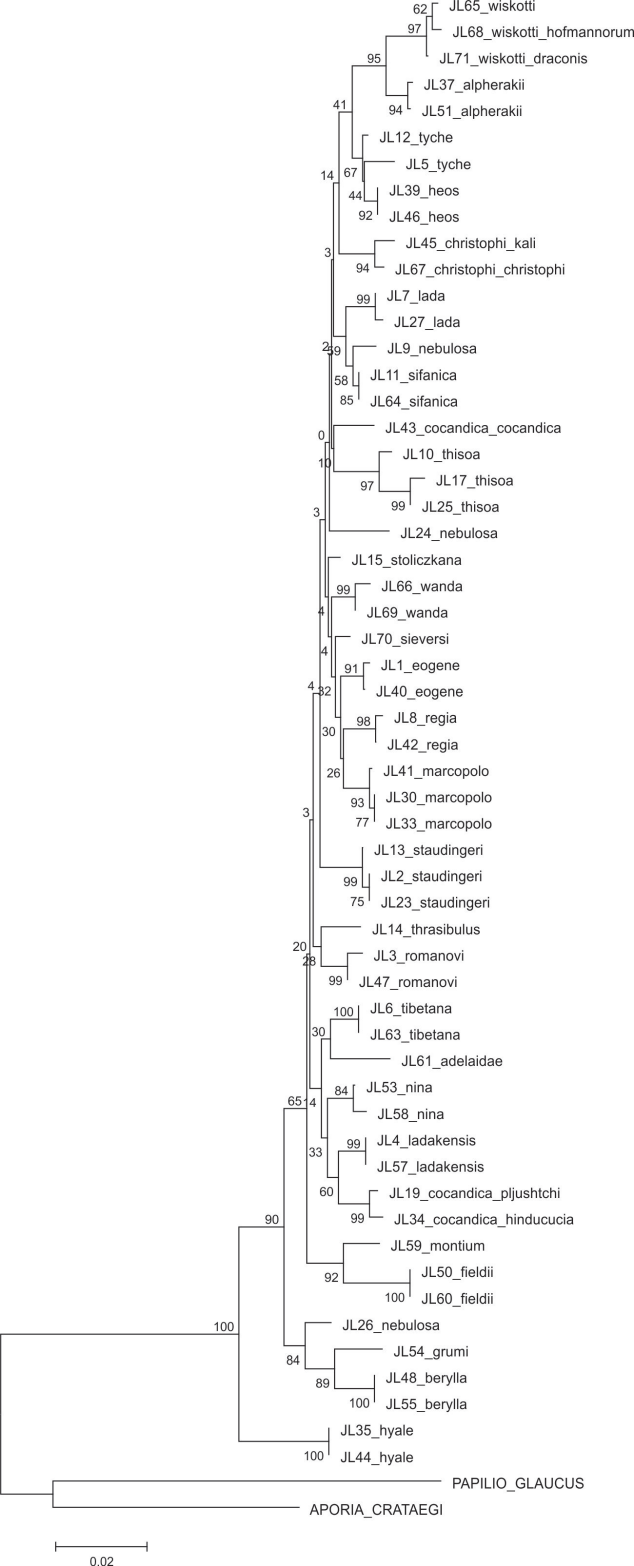
Neighbour-Joining tree using the K2P-model for the COI sequences obtained in this study.

The parsimony analysis of the new COI sequences yielded four equally parsimonious trees (CI = 0.59, RI = 0.75) the strict consensus tree of which is presented in Figure 1. The NJ tree is presented in Figure 2.

**Figure 3.**
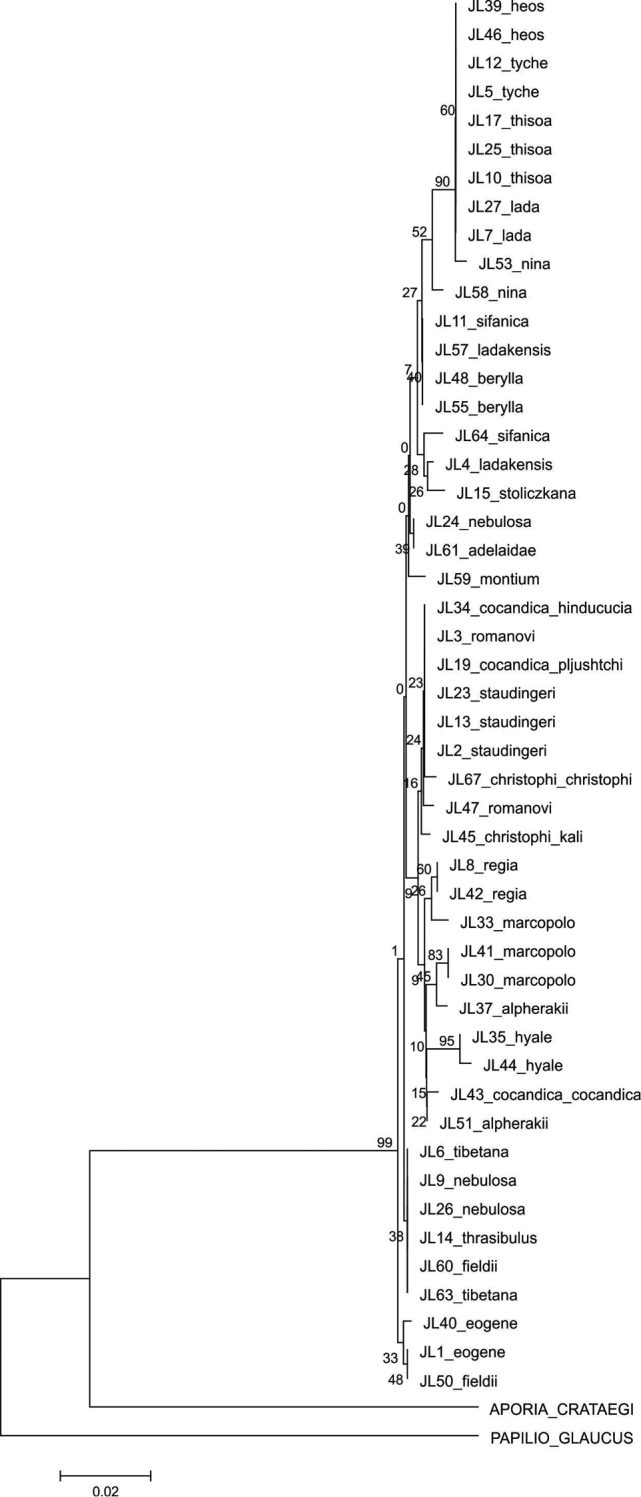
Neighbour-Joining tree using the Tamura-Nei model with gamma distributed rates for the RpS2 sequences.

The majority of the species could be identified with COI alone, as no COI haplotypes were shared between species. Both parsimony and NJ trees recovered 25 (out of 28) species as monophyletic groups (Figures 1–2). Neither *Colias cocandica*, nor *Colias nebulosa* formed monophyletic entities, as their sequences were scattered over various parts of the trees. The two samples of *Colias tyche* were not recovered as sister taxa, for sample MZH_JL5 appeared as sister taxon of *Colias heos*. The overall topologies of the parsimony and NJ trees were identical, except for the placement of *Colias thrasibulus*. Parsimony placed the taxon as sister to a clade of five taxa (Figure 1), while NJ placed it as sister to *Colias romanovi* (Figure 2). The external morphology of *Colias thrasibulus* is rather different from that of *Colias romanovi*, while some similarities can be found between *Colias thrasibulus* and *Colias nina*, *Colias ladakensis*, *Colias tibetana* and *Colias cocandica* (Figure 1). Only 17 of the 39 parsimony informative sites of RpS2 were variable among the 49 ingroup members. NJ only recovered few species as separate lineages due to the shallow divergences (Figure 3). The information content of this gene region is best interpreted as a character-based diagnostic table, as suggested by DeSalle et al. (2005). This gene region yielded species specific (diagnostic) haplotypes for 11 species out of 33 (Table 3).

**Table 3.** Species haplotypes for 17 variable positions of RpS2 for Central Asian *Colias* species (RpS2 data matrix positions no 14, 152, 170, 176, 189, 191, 194, 195, 218, 284, 287, 302, 341, 353, 356, 365, 380).

Haplotype	positions of RpS2
MZH_JL35_hyale	TCCCCGGGTCCATTTTC
MZH_JL44_hyale	TCCCCGGGTCCATTTTC
MZH_JL02_staudingeri	TCCTCGAGTTCAAATCC
MZH_JL13_staudingeri	TCCTCGAGTTCAAATCC
MZH_JL23_staudingeri	TCCTCGAGTTCAAATCC
MZH_JL43_cocandica_cocandica	TCCCCGAGTTCAAATCC
MZH_JL41_marcopolo	TACCCGAGTTCAAAACC
MZH_JL30_marcopolo	TACCCGAGTTCAAAACC
MZH_JL07_lada	TCCCAAAAGTCGATTCC
MZH_JL27_lada	TCCCAAAAGTCGATTCC
MZH_JL25_thisoa	TCCCAAAAGTCGATTCC
MZH_JL10_thisoa	TCCCAAAAGTCGATTCC
MZH_JL17_thisoa	TCCCAAAAGTCGATTCC
MZH_JL05_tyche	TCCCAAAAGTCGATTCC
MZH_JL12_tyche	TCCCAAAAGTCGTTTCC
MZH_JL39_heos	TCCCAAAAGTCGATTCC
MZH_JL46_heos	TCCCAAAAGTCGATTCC
MZH_JL53_nina	TCCCAAAAGTCGATTCC
MZH_JL58_nina	CCCCCGAAGTCGATTCC
MZH_JL11_sifanica	TCCCCGAGGTCGWTTCC
MZH_JL64_sifanica	TCTCCGAGGTCGATTCC
MZH_JL57_ladakensis	TCCCCGAGGTCGATTCC
MZH_JL06_tibetana	TCCTCGAGGTTATTTCC
MZH_JL09_nebulosa	TCCTCGAGGTTATTTCC
MZH_JL26_nebulosa	TCCTCGAGGTTATTTCC
MZH_JL14_thrasibulus	TCCTCGAGGTTATTTCC
MZH_JL01_eogene	TCCTCGAGGTTATTTCT
MZH_JL04_ladakensis	TCTCCGAGGTTATTTCC
MZH_JL15_stoliczkana	TCTCCGAGGTTGTTTCT
MZH_JL19_cocandica_pljushtchi	TCCTCGAGTTCATTTCC
MZH_JL34_cocandica_hinducucia	TCCTCGAGTTCATTTCC
MZH_JL03_romanovi	TCCTCGAGTTCATTTCC
MZH_JL08_regia	TCCCCGAGTTCATTTCT
MZH_JL42_regia	TCCCCGAGTTCATTTCT
MZH_JL47_romanovi	CCCTCGAGTTCATTTCC
MZH_JL51_alpherakii	TCCCCGAGTTCATTTCC
MZH_JL37_alpherakii	CACCCGAGTTCATTTCC
MZH_JL67_christophi_christophi	TCCTCGAGTTCATTTCC
MZH_JL45_christophi_kali	TCCTCGAGTTCGTTTCC
MZH_JL40_eogene	TCCTCGAGGTTGTTTCT
MZH_JL24_nebulosa	TCCTCGAGGTCGTTTCC
MZH_JL59_montium	CCCTCGAGGTTGTTTCC
MZH_JL61_adelaidae	TCCTCGAGGTCGTTTCC
MZH_JL60_fieldii	TCCTCGAGGTTATTTCC
MZH_JL50_fieldii	TCCTCGAGGTTATTTCT
MZH_JL33_marcopolo	TCCCCGAGGTCATTACT
MZH_JL63_tibetana	TCCTCGAGGTTATWTCC
MZH_JL48_berylla	TCCCCGAGGTCGAATCC
MZH_JL55_berylla	TCCCCGAGGTCGAATCC

### Analysis of the concatenated COI + RpS2 data

**Figure 4.**
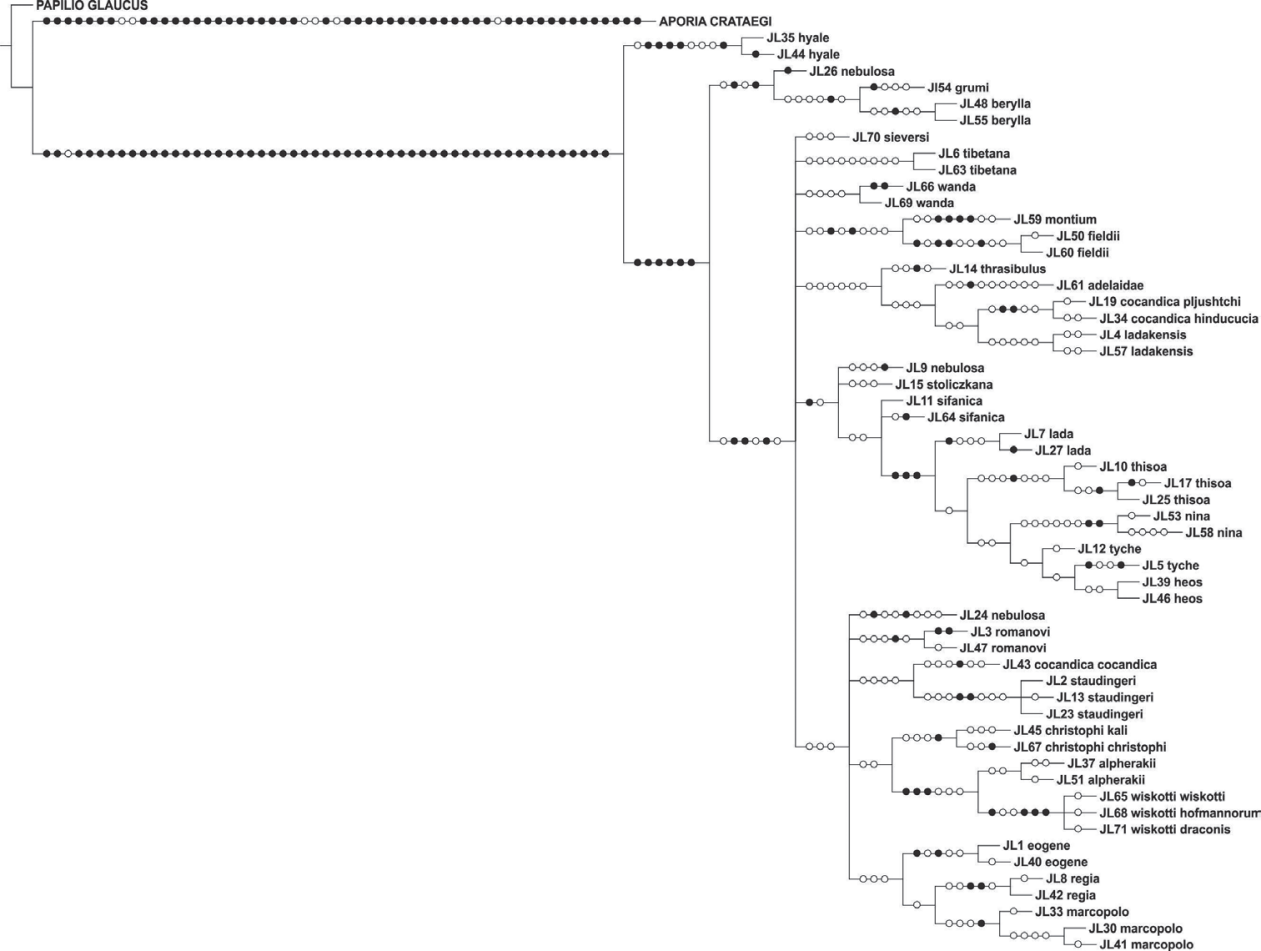
Strict consensus cladogram of the concatenated data set of COI + RpS2.

The parsimony analysis of COI + RpS2 yielded nine trees of length 560 steps (CI = 0.63, RI = 0.72), the strict consensus tree of which is shown in Figure 4. *Colias cocandica*, *Colias nebulosa* and *Colias tyche* were not monophyletic and *Colias thrasibulus* had the same position as in the COI cladogram (Figure 1).

### Analysis of all the COI sequences

The strict consensus cladogram for all the available COI data resolved the taxa in the same positions as in the tree of the new COI sequences only. For ten species of the present study sequences were also available from GB. Sequences of most species clustered together as monophyletic entities, except for *Colias nebulosa*, *Colias cocandica*, *Colias tyche* and *Colias regia*. For *Colias regia* the GB sequence (GB accession no FJ663427) did not cluster together with our sequences. The GB barcodes of *Colias erate* and *Colias croceus* were shared by these two taxa.

Neither the Himalayan and south Tibetan adjacent mountain *Colias* fauna (*Colias berylla*, *Colias ladakensis*, *Colias nina*, *Colias stoliczkana*, *Colias thrasibulus*, *Colias tibetana*), nor the east Tibetan, Qinghai, Gansu and Sichuan species aggregates (*Colias adelaidae*, *Colias grumi*, *Colias lada*, *Colias montium*, *Colias nebulosa*, *Colias sifanica*, *Colias wanda*) were resolved as species clusters similar to the Tian Shan, Pamir and Hindukush species.

Several COI haplotypes were noted for a few species, even among specimens obtained from the same locality (e.g. *Colias staudingeri* and *Colias thisoa*). Taxa not resolved as monophyletic clusters were the species *Colias cocandica* and *Colias nebulosa*. All the included subspecies of *Colias cocandica* (*Colias cocandica cocandica*, *Colias cocandica pljutshtshi* and *Colias cocandica hinducucia*) showed distinct COI sequences, with *Colias cocandica cocandica* as most different.

## Discussion

### Barcoding

Lukhtanov et al. (2009) tested the utility of COI barcodes for Central Asian butterflies by sampling specimens from a considerable geographical range. They observed that this substantially increased intraspecific variation reducing the interspecific divergences (“barcoding gap”), but that this did not hamper species identification. The present study shows that most *Colias* taxa form monophyletic entities that can be identified with COI data alone. The RpS2 gene region showed identical sequences in *Colias cocandica pljutshtshi* and *Colias cocandica hinducucia* (Table 3, Figure 3), differing by only three nucleotides from *Colias cocandica cocandica*. Based on the molecular data the recognition of these subspecies is not or weakly supported.

The fact that the three *Colias nebulosa* samples were scattered over different parts of the COI tree might be the result of a laboratory contamination due to carry over between samples. The *Colias nebulosa* samples were collected on the same day and in the same place. *Colias nebulosa* is morphologically distinct from other *Colias* species, excluding possible misidentification. The RpS2 data, however, could point to two morphologically cryptic species in sympatry (samples MZH_JL24 vs. MZH_JL9 and MZH_JL26), so that the different COI barcodes might represent numts, despite no apparent ‘signs’ (no indels). This discrepancy between morphology and DNA sequence data emphasises the necessity to use multiple samples to detect this sort of challenging issues.

Even though *Colias cocandica* and *Colias nebulosa* did not form monophyletic groups our results show that COI barcodes are useful for (1) identifying Palaearctic and Central Asian *Colias*, (2) pointing to a possible cryptic species, and (3) highlighting the necessity to further investigate the question on the subspecific rank of *Colias cocandica cocandica*.

The utility of RpS2 as a species barcode for *Colias* spp. is clearly more limited, since e.g. *Colias heos*, *Colias lada*, *Colias nina*, *Colias thisoa* of the subgenus *Eriocolias* and *Colias tyche* (subgenus *Eucolias*) have identical sequences (Table 3, Figure 3). Still, RpS2 yielded species specific (diagnostic) haplotypes for 11 species of the subgenus *Eriocolias* and for *Colias hyale* (subgenus *Colias* s.str.).

### Congruence with traditional classification: analysis of concatenated COI + RpS2

The strict consensus tree was more resolved than either of the trees resulting from separate analyses of the gene regions (Figure 4).

Although the concatenated data did not resolve the phylogenetic relationships among all *Colias* species, some observations can be made. The majority of the species confined to the adjacent Tian Shan, Pamir and Hindukush mountain ranges form a well supported clade. This includes *Colias eogene*, *Colias regia*, *Colias romanovi*, *Colias marcopolo*, *Colias staudingeri*, *Colias christophi*, *Colias alpherakii* and *Colias wiskotti*. Yet, *Colias sieversi*, which also occurs in these mountain ranges (Peter I and Khozratishoh mountains), was not included in this clade. *Colias sieversi* is morphologically most similar to *Colias alpherakii*, thus showing another case of disagreement between morphological and DNA sequence data. *Colias thisoa*, too, lives in the aforementioned mountain ranges, but it has a wider distribution, stretching from Turkey to the Altai Mountains. A third taxon, *Colias cocandica cocandica*, is considered closely related to *Colias tamerlana* (e.g. Verhulst 2000), a species occurring in southern Siberia and Mongolia. Thus, the origin of *Colias thisoa* and *Colias cocandica cocandica* may differ from that of the species confined to the Tian Shan, Pamir and Hindukush mountain range. One sample of *Colias cocandica* (MZH_JL43) was placed within this “mountain” clade, while the other two samples appeared as sister taxa to the Himalayan species *Colias ladakensis*. As with *Colias sieversi*, our DNA data disagree with the morphological characters, but it should be noted that this clade is not well supported. Conversely, two morphologically similar Himalayan species, viz. *Colias nina* and *Colias ladakensis*, were assigned to different clades. In the COI + RpS2 tree they were placed in different, more encompassing species clusters (Figure 4), in the COI NJ tree they were joined with *Colias cocandica pljutshtshi* and *Colias cocandica hinducucia* (Figure 2), while the COI cladogram resolved these taxa together with *Colias adelaidae*, *Colias tibetana*, *Colias cocandica pljutshtshi* and *Colias cocandica hinducucia* (Figure 1).

The analyses did not support the monophyly of the subgenera *Eucolias* and *Eriocolias* sensu Berger (1986). The *Eucolias* species *Colias tyche* was not resolved as a separate monophyletic lineage, but was resolved into *Eriocolias*. This is congruent with the results of Pollock et al. (1998) and Brunton (1998). Only the the subgenus *Colias*, here represented by *Colias hyale*, is supported as a distinct lineage, placed as sister to all other *Colias* sp.

### Barcodes of Palaearctic *Colias* spp.

**Figure 5.**
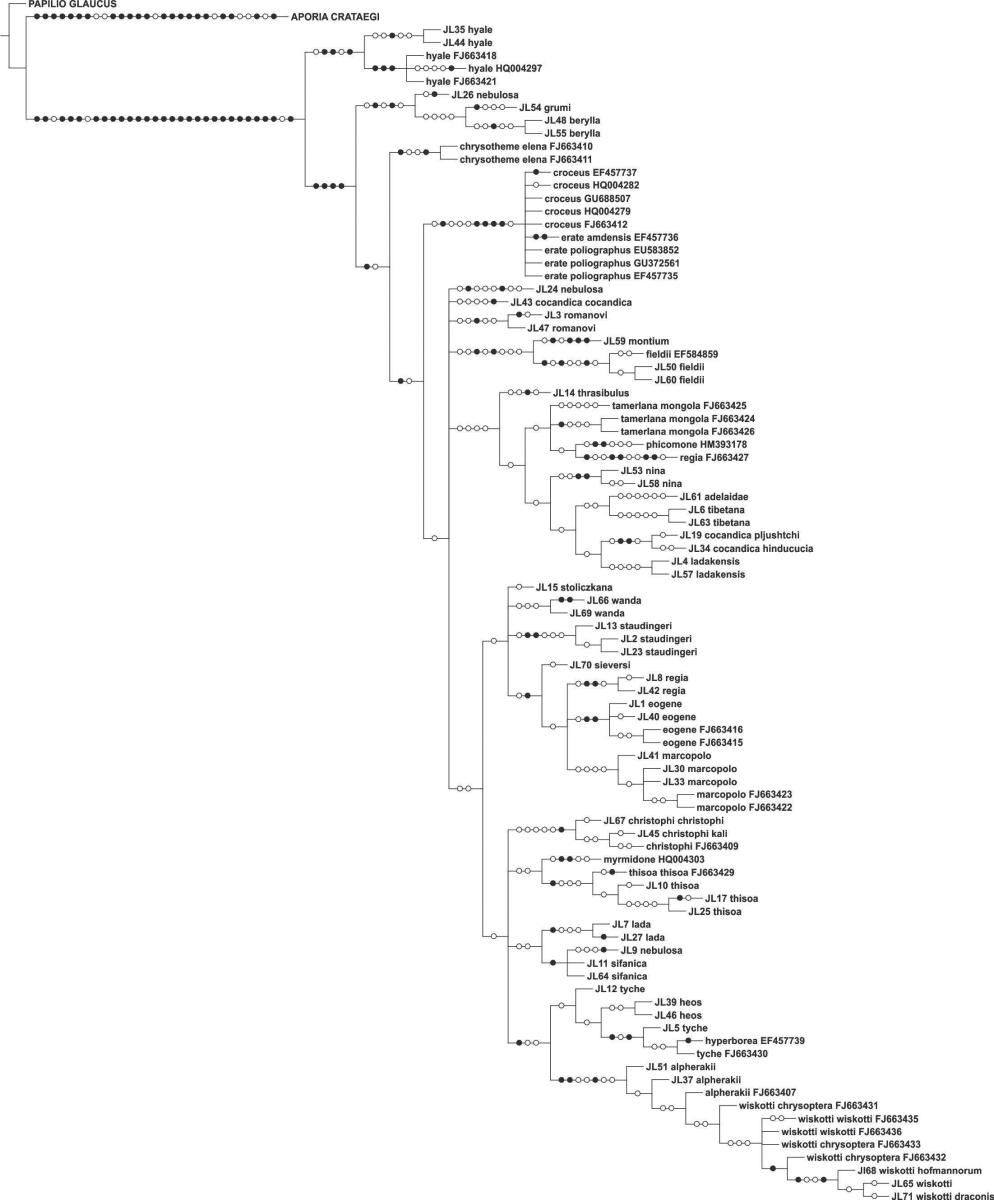
Strict consensus cladogram of COI sequences for Palaearctic *Colias* taxa.

**Figure 6.**
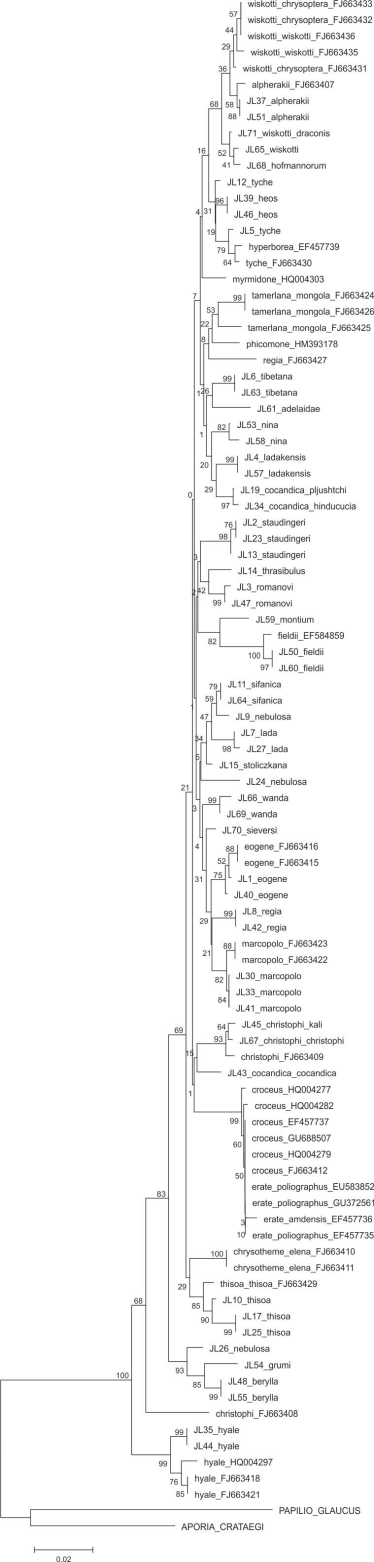
Neighbour-Joining tree using the K2P-model of COI sequences for Palaearctc *Colias* taxa.

The parsimony (Figure 5) and NJ analyses (Figure 6) of the larger matrix of Palaearctic COI barcodes (total COI) recovered the same species clusters, but some of the species show different placements (e.g. *Colias thisoa*, *Colias christophi*). This is not surprising as all internal nodes are very shallow. The samples of *Colias tyche* and *Colias hyperborea* show very low sequence difference, morphologically these taxa are different, and they largely share the same distribution area. An example of species that share the same distribution and that exhibit clear morphological similarities, and which as such were resolved as sister species in both analyses, includes *Colias wiskotti* and *Colias alpherakii*. Identification of Palaearctic *Colias* based on COI barcodes is in most cases possible, since shared haplotypes were recorded only for *Colias erate* and *Colias croceus*.

Intraspecific variation is notable between some of the recognized subspecies, both among our own samples and those downloaded from GB. The intraspecific variation can partly be explained by morphologically clearly distinct subspecies, such as those of *Colias wiskotti*, or by specimens from widely different localities, such the different specimens of *Colias hyale* (sample FJ663418 from Russia, FJ663421 from Kazakhstan, HQ004297 from Romania and MZH_JL35 and MZH_JL44 from SW Transbaikalia). However, notable intraspecific variation also occurs within populations, such as *Colias thisoa aeolides* with all samples originating from the same locality and date, but the limited sampling prevents conclusions on the reasons for this. It is apparent that the understanding of intraspecific variability of the COI barcode for *Colias* is presently very limited.

The combined COI data of our sequences and sequences downloaded from GB include species belonging to one additional subgenus, *Neocolias*, represented by *Colias myrmidone* and *Colias erate*. Only the subgenus *Colias*, represented by *Colias hyale*, is well supported as distinct lineage. Yet, one specimen of *Colias hyale* (FJ663419) clustered together with *Colias erate* (*Neocolias*) and *Colias croceus* (*Eriocolias*). The other subgenera were not resolved as clades according to present classification, in agreement with our results for the combined analysis.

Our findings generally support COI as a species specific barcode for *Colias*, but we also highlight the necessity of including multiple individuals of species in molecular barcoding studies. Problematic ‘cases’ of widely divergent barcodes or conflicting morphological and molecular ‘signals’ are found in most if not all barcoding studies, and this study makes no exception.
